# Species specificity and sexual dimorphism in tooth shape among the three sympatric haplochromine species in Lake Kivu cichlids

**DOI:** 10.1002/ece3.6309

**Published:** 2020-05-14

**Authors:** Philippe Munyandamutsa, Wilson Lazaro Jere, Daud Kassam, Austin Mtethiwa

**Affiliations:** ^1^ Africa Centre of Excellence in Aquaculture and Fisheries Department of Aquaculture and Fisheries Science Bunda College Lilongwe University of Agriculture and Natural Resources (LUANAR) Lilongwe Malawi; ^2^ Department of Animal Production College of Agriculture, Animal Sciences and Veterinary Medicine University of Rwanda (UR) Musanze Rwanda

**Keywords:** elliptic fourier analysis, *Haplochromis*, Lake Kivu, tooth shape

## Abstract

Tooth shape is used to differentiate between morphologically similar species of vertebrates, including fish. This study aimed to quantify tooth shape of three sympatric species: *Haplochromis kamiranzovu*, *H. insidiae*, and *H. astatodon* endemic to Lake Kivu, whose existing identification criteria are currently only qualitative. A quantitative tooth shape analysis was performed based on digitized tooth outline data with a subsequent elliptic Fourier analysis to test for differences among the three species. We looked at crown shape and size differences within *H. kamiranzovu* and *H. insidiae* at geographical, habitat, and gender levels. No comparison at habitat level was done for *H. astatodon* because it is found only in littoral zone. The analysis revealed significant tooth shape differences among the three species. *Haplochromis astatodon* had a significantly longer major cusp height and a longer and larger minor cusp than that of *H. insidiae.* It had also a longer major cusp height and a longer and larger minor cusp than that of *H. kamiranzovu*. Tooth shape differences of *H. kamiranzovu* and *H. insidiae* species were not significantly different between littoral and pelagic fish (*p* > .05) while differences were significant between southern and northern Lake Kivu populations (*p* < .05). Tooth sizes in *H. kamiranzovu* and *H. insidiae* were significantly different, both in height and width as well as in their ratios, and this was true at sex and geographic levels (*p* < .05), but not at habitat level (*p* > .05). Tooth shape was also significantly different with sharp teeth for males compared with females of southern populations versus northern ones. These shape‐ and size‐related differences between sexes suggest differences in the foraging strategies toward available food resources in the lake habitat. Further research should explain the genetic basis of the observed pattern.

## Graphical abstract

We quantified tooth shape trait to distinguish the three morphological similar *Haplochromis kamiranzovu*, *H. insidiae*, and *H. astatodon*) endemic to Lake Kivu.

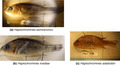

## 1 INTRODUCTION

Tooth shape is often used as a taxonomic tool in vertebrates. For example, the enamel–dentine junction morphology and enamel thickness in early hominid molars proved to be useful for taxonomic studies (Ayvazyan, Vasilyan, & Böhme, 2019; Delgado et al., 2015) and the cranial dental morphology was used to assess the taxonomic status of the hoolock species in China (Fan et al., 2017). Crown height and crown base were found to be key identification features for different phytosaur teeth from the upper Triassic of India (Datta, Kumar, & Ray, 2019) and the upper molar morphology appeared to be taxonomically diagnostic within the genus Pongo (Ortiz et al., 2019).

Although tooth shape is considered to be under strict genetic expression (Hallikas et al., 2019; Ramya, Priya, & Gayathri, 2019; van Rijssel et al., 2015), it is not always an appropriate species delimiter. Its involvement in feeding makes it an important structure that is often under selection, especially during adaptive radiations that are triggered by shifts in trophic ecology, as is the case in cichlids fishes from East Africa (Ahi, Duenser, Singh, Gessl, & Sturmbauer, 2019; Futuyma, 1986; Greenwood, 1974; Ribbink, Marsh, Marsh, Ribbink, & Sharp, 1983; Ruber, Verheyen, & Meyer, 1999). Tooth shape frequently reflects trophic niches that are being explored (Fitzgerald, Winemiller, Sabaj Pérez, & Sousa, 2017; Streelman, Webb, Albertson, & Kocher, 2003). As tooth shape becomes quickly adapted to the different habitats these fishes occupy (Raia, Carotenuto, Meloro, Piras, & Pushkina, 2010), it frequently reflects the type of food a species has become adapted to consume (Davit‐Béal, Tucker, & Sire, 2009; Evans, 2013; Huysseune & Sire, 1998; Pasco‐Viel et al., 2010; Streelman et al., 2003; Takahashi, Watanabe, Nishida, & Hori, 2007), even within a single generation (Calandra, Labonne, Schulz‐Kornas, Kaiser, & Montuire, 2016; French et al., 2017). Several other phenotypic traits have shown functional divergence under ecological disparity, such as the pharyngeal jaw (Magalhaes, Ornelas‐Garcıa, Leal‐Cardin, Ramírez, & Barluenga, 2015), the gill rakers (Schluter & McPhail, 1992), body shape (Barluenga, Stölting, Salzburger, Muschick, & Meyer, 2006), gut length (Wagner, McIntyre, Buels, Gilbert, & Michel, 2009), mouth position (Burress, Holcomb, & Armbruster, 2016), snout length (Bonato, Burress, & Fialho, 2017), fin length, and body size (Farré, Tuset, Maynou, Recasens, & Lombarte, 2013), in the same line like as tooth size and shape. These traits indicate a fish species’ function in an aquatic ecosystem where it occurs (Córdova‐Tapia, Contreras, & Zambrano, 2015).

Tooth shape has also been used to detect differences among species (Ayvazyan et al., 2019; Ikejiri & Lucas, 2014; Ruber et al., 1999) and variations within individuals of the same species (Streelman, 2003). The taxonomic value of tooth shape as a usable diagnostic trait depends on the level of intraspecific variation compared to possible interspecific differences. Taxonomic studies on cichlids generally rely on tooth shape as a characteristic to differentiate among species (Ayvazyan et al., 2019; Carpenter, Miles, & Cloward, 1998; De Zeeuw, Westbroek, Oijen, & Witte, 2013; Sereno et al., 1999; Snoeks, 1994; Wautier, Huysseune, & Verheyen, 2002). For example, among Lake Victoria cichlids, having unicuspid and obliquely truncated teeth is one of the most distinctive features to identify *Haplochromis obliquidens* Hilgendorf, 1888 (Witte & Oijen, 1990). However, the precise characterization of the species‐specific differences sometimes remains ambiguous. In Lake Kivu, identification of *Haplochromis astatodon* Regan, 1921, *H. insidiae* and *H. kamiranzovu* Snoeks, 1987 proved to be very difficult. This confusion is due to the fact that some specimens of *H. kamiranzovu* have a tooth profile that might be confused with that of the somewhat truncated teeth with an expanded crown found in *H. insidiae* (Snoeks, 1994). For *Haplochromis astatodon*, it was noted that this species can unambiguously be distinguished from all other Lake Kivu haplochromines except *H. insidiae* by the characteristic shape of its outer oral jaw teeth. Teeth of *H. astatodon* are more oblique and also more truncated than those from *H. insidiae* (Snoeks, 1994), but without a clear difference between them. In this scenario, the use of outlines, combined with an Elliptic Fourier Analysis (EFA) would show clear differences in tooth shape between *Haplochromis astatodon*, *H. insidiae* and *H. kamiranzovu*.

In another study, tooth shape was linked to sexual differences (Hulsey, García‐De León, & Meyer, 2015; Mallon, 2017). For example, sex‐specific differences in tooth shape were found in the Jordan Lake, *Haplochromis flaviijosephi*. Adult males have conical teeth while females have bicuspid teeth (Spataru & Gophen, 1985).

Environmental conditions have also been hypothesized to drive the evolution of numerous trophic morphologies (Dieleman, Bocxlaer, Nyingi, Lyaruu, & Verschuren, 2019; Herler, Kerschbaumer, Mitteroecker, Postl, & Sturmbauer, 2010; Streelman & Albertson, 2006; Takashi & Koblmüller, 2011). For example, there were environmentally induced differences in teeth morphology between males and females in cichlid fish (Cichlidae) of Lake Tanganyika (Yamaoka, 1983) and in a sockeye salmon population (Salmonidae) in Pacific Ocean (Johnson, Carlson, & Quinn, 2006) which showed that within each of their breeding habitats, males had longer teeth than females.

Dentition was also found to be an excellent indicator of feeding type in cichlid fishes (Albertson & Kocher, 2006; Hulsey et al., 2006). Considering local variation in niche traits within a lake environment, studying tooth morphology of specimens from heterogeneous local habitats might reflect micro‐level trophic variation. For example, fish from pelagic and littoral habitats in Lake Kivu, Rwanda, were found to represent two distinct environments (Masilya, Darchambeau, Isumbisho, & Descy, 2011). In Lake Kivu, littoral substrates comprise submerged rocks, macrophytes, and sandy, muddy, rock‐sandy, and mixed habitats where zooplankton density is 15–50 times lower than in the pelagic zone (Isumbisho, Kaningini, Descy, & Baras, 2004; Isumbisho, Sarmento, Kaningini, Micha, & Descy, 2006). The coastal area is abundant with *Cladophora* on the rock surface (Verbeke, 1957) and nauplii (Isumbisho et al., 2006), while the pelagic zone is dominated by a complex microbial community, including bacteria and phytoplankton (with taxa like *Nitzschia bacata* and *Fragilaria danica* and cyanobacteria like *Planktolyngbya limnetica* and *Synechococcus* sp., Wimba, 2009; Sarmento et al., 2007). In the pelagic zone, zooplanktons are composed of three most abundant species of adult copepods (*Mesocyclopsa equatorialis* Kiefer 1929, *Thermocyclops consimilis* Kiefer 1934 and *Tropocyclops confinis* Kiefer 1930), four species of cladocera (*Diaphanosoma excisum* Sars 1885, *Coronatella rectangular* Sars 1861, *Ceriodaphnia cornuta* Sars 1885, *Moina micrura* Kurz 1875) and five most common species of rotifers (Bdelloids, *Keratella tropica* Apstein 1907, *Lecane* sp., *Brachionus* sp., *Anuraeopsis fissa* Gosse 1851) (Isumbisho et al., 2006).

The adaptive value of tooth size in the three Lake Kivu haplochromines has not yet been studied in relation to the habitat (littoral vs. pelagic). Studying tooth size variation of the haplochromines species offers an opportunity to explore the relationship between crown size and habitat gradient from the littoral versus pelagic zone haplochromines of Lake Kivu.

We tested the hypothesis that tooth shape indeed varies among *Haplochromis astatodon*, *H. insidiae* and *H. kamiranzovu,* and hence is a taxonomically reliable trait among these Lake Kivu cichlids. We also examined the hypothesis that tooth size vary among *Haplochromis astatodon*, *H. insidiae* and *H. kamiranzovu*.

Specifically, we examined whether *Haplochromis astatodon*, *H. insidiae*, and *H. kamiranzovu* can be identified using tooth shape, and whether tooth size (i.e., height and width) of major cusps can be another tool to aid in diagnosis of the three species. We also tested for tooth shape differences between sexes, habitat, and region in major cusp heights and widths of the three species.

## 2 MATERIALS AND METHODS

### 2.1 Study area and specimen preparation

**FIGURE 1:**
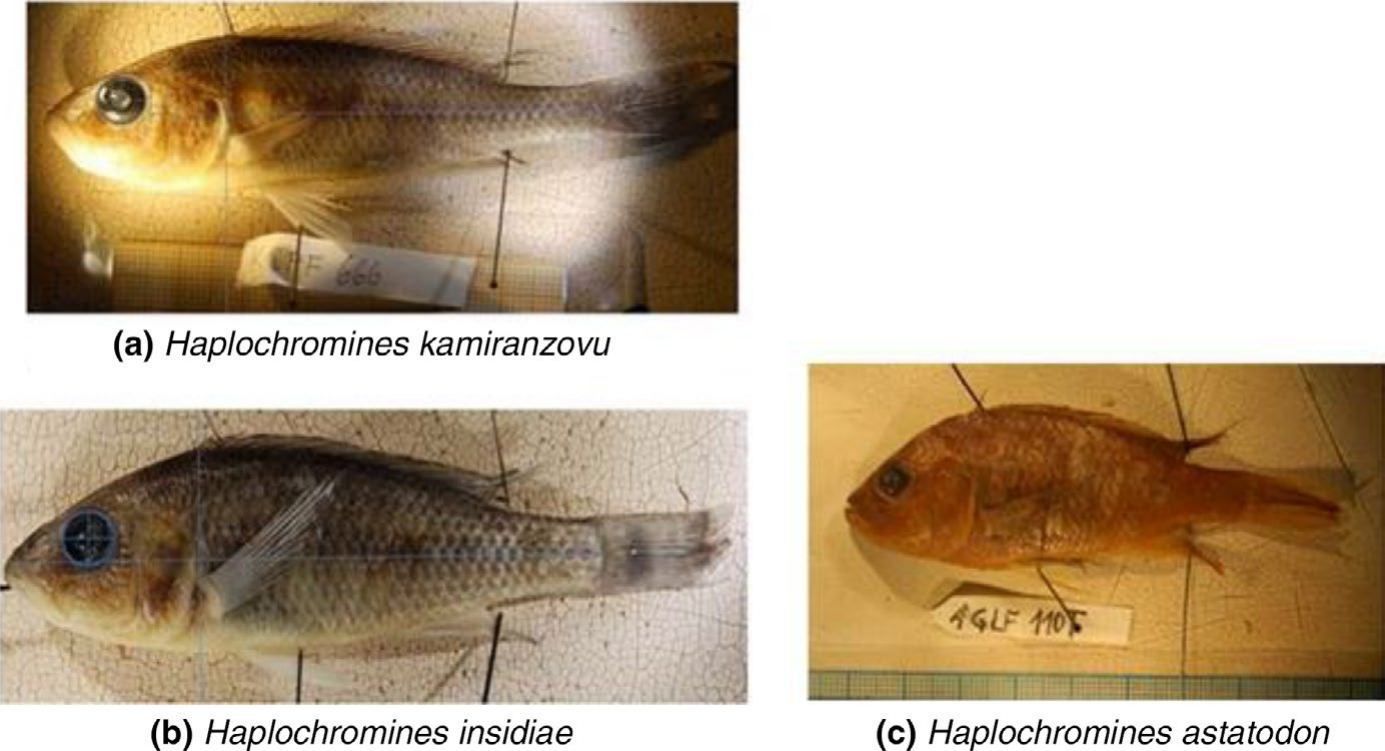
*Haplochromines* species studied. (The above are the photographs of the organisms studied)

**FIGURE 2:**
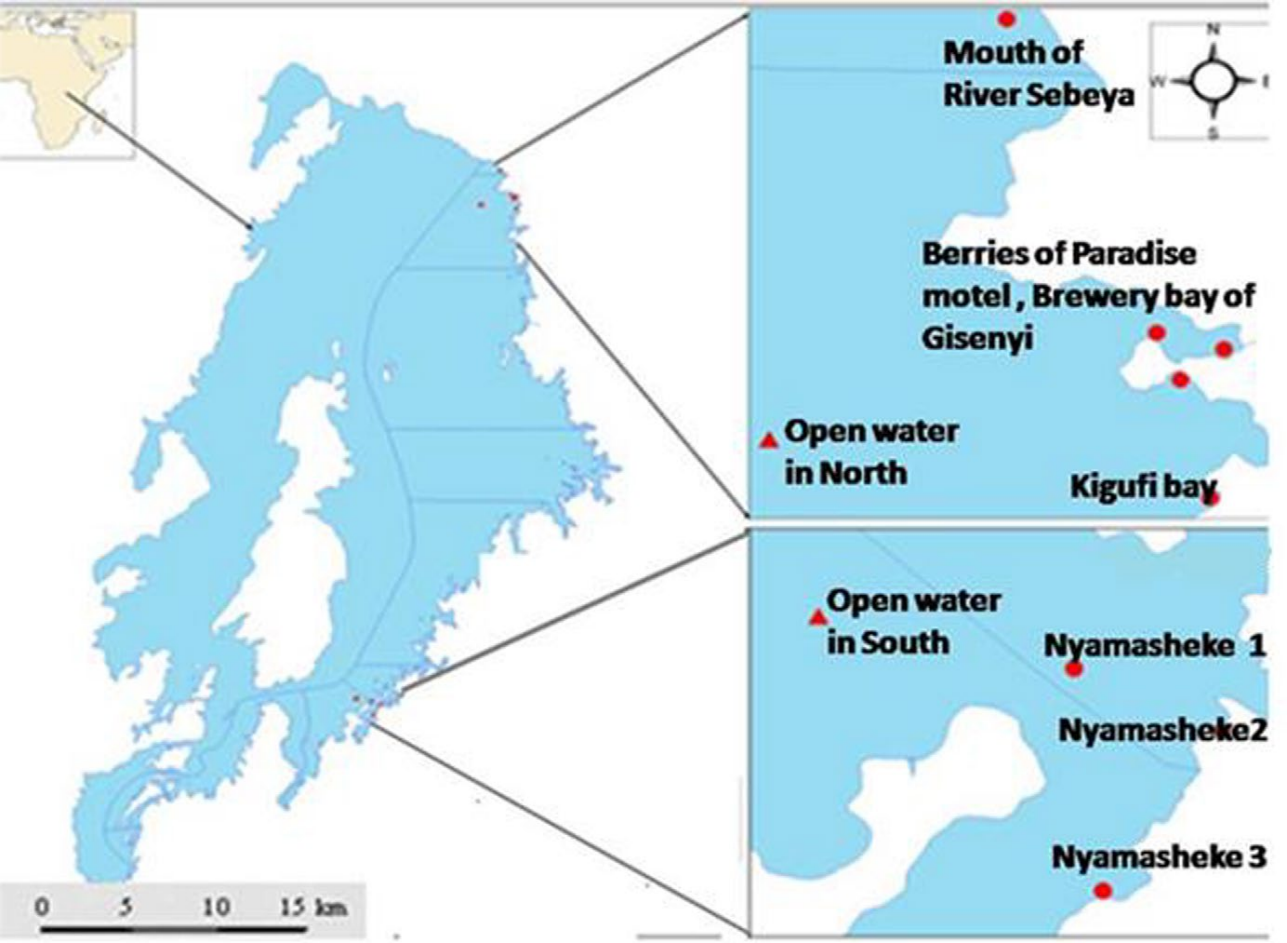
Map of Lake Kivu showing the fish sampling locations. Dots represent littoral sampling locations, and triangles represent pelagic sampling locations. Blue lines represent the expansion of the sampling area; northern and southern regions. GPS coordinates of Nyamasheke 1 are closer to an island of <40 m depth. Other GPS coordinates of Nyamasheke sampling locations are closer to the coastal zone

Teeth were obtained from adult fish (Figure 1) caught using 15 × 1 m gillnets made from monofilament nylon (10 mm mesh size) in littoral and pelagic zones of northern and southern regions of Lake Kivu. In the north, we caught fish from the Brewery bay of Gisenyi, Berries of Paradise motel, Kigufi bay, and Mouth of Sebeya River. After several unfruitful fishing of the targeted species along the south shore, sampling was systematically done only at three sites where both haplochromines species occurred in Nyamasheke (Nyamasheke 1, Nyamesheke 2, and Nyamasheke 3). These sampling locations were chosen in order to get a disparate representation of tooth shape variation across the lake. In addition, one sampling was done in the open waters of the northern part of the lake and another sampling was done in the open waters of the southern part (Figure 2). Haplochromines samples were collected during the dry season (from June to September 2018). Since the haplochromine species we investigated are very rare (Kaningini et al., 1999), fish samples were collected during early morning, late morning, and in the afternoon (05 00–07 00 hr; 10 00–12 00 hr and 15 00–17 00 hr) using eight mm mesh nets (15 × 2 m) to get a representative number of fish samples for statistical analyses.

These are periods of intense activity of *H. kamiranzovu* (Ulyel, Ollevier, Ceuster, & Audenaerde, 1990). On the first days of the month, only littoral samples were collected in the morning, late morning, and in the afternoon, while pelagic samples were collected at night. The easy way to get the haplochromines in the pelagic zone was to follow *Limnothrissa miodon* fishermen at night when they are allowed to operate. At night, nets were set to a maximum depth of 80 m in the pelagic zone once a month. The night samples were also collected at the following times: 21 00–22 00 hr; 23 00–01 00 hr; and 02 00–05 00 hr.

*Haplochromis insidiae* and *H. kamiranzovu* contribute 0.01% and 16.62% of total fish catches in littoral zone of Lake Kivu, respectively (Kaningini et al., 1999). No catch statistics have been reported for *H. astatodon*, which is known only from a specific littoral habitat. We did not catch any *H. astatodon* specimens in our 3 months of fish sampling campaigns, and we thus borrowed specimens from Institut de Recherche Scientifique et Technologique (IRST), Rwanda in collaboration with Royal Museum for Central Africa (RMCA).

The fish were identified under a binocular microscope (at the magnification of 10 × 4) based on quantitative and qualitative characteristics described by Snoeks (1994), which are overall tooth shape, body shape, caudal peduncle length, and number of gill rakes. Fish were given an identification number (ID) indicating sex and sampling location.

Immediately after identification of the fish at the capture site, formalin solution (5%) was injected into the fish esophagus. Thereafter, the whole fish was submerged in formalin solution (5%). Fish remained in this solution for 2 weeks in the Ichthyology Laboratory of the Bio‐Technology Complex at the University of Rwanda. After 2 weeks, fishes were washed overnight with running water, then transferred into alcohol of 30%, 60% then in 70% as recommended by Wanink, Witte, and Kishe‐Machumu (2008). After that, they were cleared and stained according to Potthoff (1984); Taylor & Van Dyke protocol (1985). The Alcian blue gives the cartilage a blue hue and Alizarin Red S, a red dye, acts on the bone. Clearing and staining was done in 10 consecutive steps: (a) dissection and removal of skin, (b) removal of the gastrointestinal track and gonads, (c) dehydration in 95% ethanol, (d) placement of the fish into Alcian blue staining for cartilage, (e) neutralization, (f) bleaching the specimens in 15% hydrogen peroxide and 85% potassium hydroxide solution, (clearing step 1), (g) staining for bone in Alizarin solution, (h) placement of the specimens into trypsin solution (clearing step 2), (i) putting the specimens into glycerine, and (j) removal of the jaw bones from the skull and labeling them with the same IDs as the specimen from which they were removed. The dentaries and premaxillae were dissected and stained using an Alizarin red S solution, and soft tissues were removed by maceration in 0.5% of KOH following Wautier et al., (2002). The teeth were then extracted with tweezers from the jaws of adult fish of *H. kamiranzovu*, *H. insidiae*, and *H. astatodon* species. Teeth were labeled with the same ID as the fish from which they were extracted.

The teeth from the collection of the IRST in collaboration with RMCA were also included and are referred to as IRST group and taken as part of our samples. The IRST group had a similar tooth shape with the recently collected specimens despite the time factor of their sampling period. The purpose was to increase the teeth sample size. The *H. astatodon*, *H. insidiae*, and *H. kamiranzovu* specimens of the IRST group were collected during different sampling campaigns in 1984 and 1987 in the littoral zone of Lake Kivu (Snoeks, 1994). The sex ratio of the specimens from our fish sample and the IRST group are reported in Table 1.

**TABLE 1:** Overview of specimens and teeth numbers used in this study for Elliptical Fourier Analysis, with reference to species, habitat, and sex of the specimen

OTU	IRST/RMCA group		Our sample		Total	
	Number of fish	Number of teeth	Number of fish	Number of teeth	Number of fish	Number of teeth
ANFL	3	12	–	–	3	12
ANLM	3	8	–	–	3	8
ISLF	2	6	4	4	6	10
ISLM	3	5	–	–	3	5
ISPF	–	–	–	–	–	–
ISPM	–	–	1	2	1	2
INLF	1	3	1	2	2	5
INLM	–	–	–	–	–	–
INPF	–	–	2	2	2	2
INPM	–	–	–	–	–	–
KSLF	–	–	–	–	–	–
KSLM	–	–	2	4	2	4
KSPF	–	–	–	–	–	–
KSPM	–	–	5	5	5	5
KNLF	9	10	3	3	12	13
KNLM	–	–	8	10	8	10
KNPF	–	–	4	6	4	6
KNPM	–	–	1	1	1	1
Total	21	41	31	42	52	83

Abbreviations: A, *H. astatodon*; F, Female; I, *H. insidiae*; K, *H. kamiranzovu*; L, littoral; M, Male; N, north; OTU, Operational Taxonomic Unity; P, pelagic; RMCA, Royal Museum for Central Africa; S, South.

There were 48 bicuspid teeth extracted from females and 35 from males. Fish specimens used in this study were stored individually in 70% ethanol in the Bio‐Technology Complex of University of Rwanda.

### 2.2 Dissection and image acquisition

**FIGURE 3:**
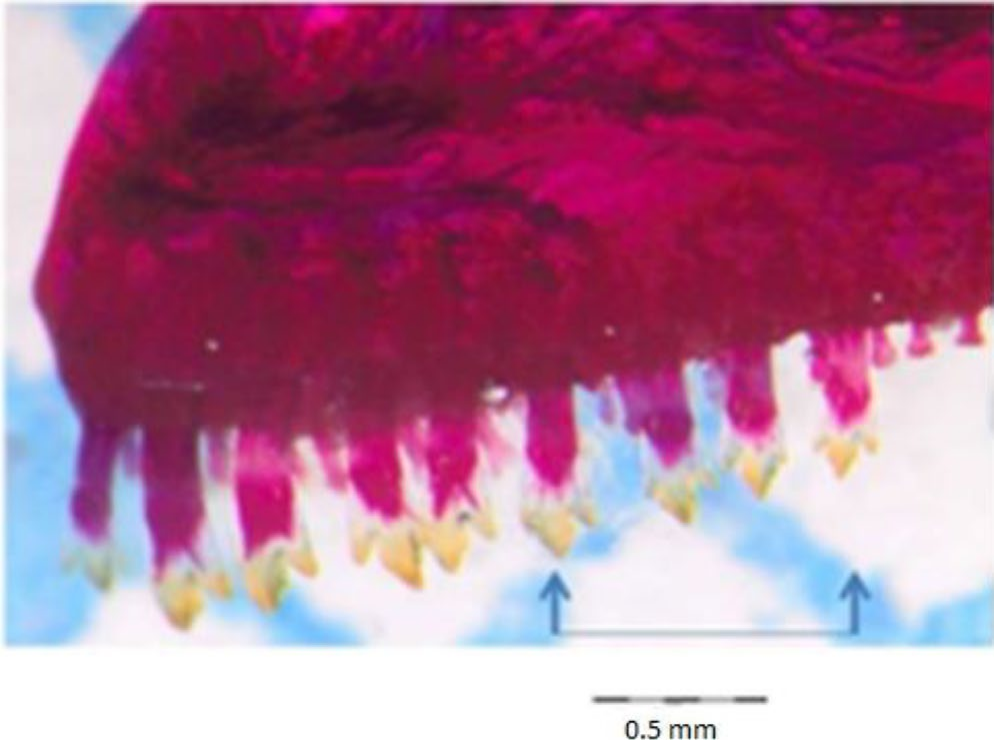
Dentition of the premaxillary in *Haplochromis kamiranzovu*. The teeth were extracted from the zone between the two arrows

A maximum number of undamaged teeth were carefully extracted at the median part of the premaxillary bone using a fine pin. Teeth were selected from this median part as earlier observations during species identification of the specimens showed that this median part, in most of the cases, bore undamaged teeth. Another reason was that at this part of the premaxillary, the teeth were found to display a consistent shape trait that facilitated species identification (Figure 3). Unfortunately, their size and positions on the premaxillary were not consistent. The tooth position trait analysis was not considered for haplochromine differentiation. That is why, even its variance within individuals of each species was not considered for the analysis. However, we performed the crown size measurements and the values obtained were normalized to the standard length of the fish.

Extraction of a complete tooth was done under an Olympus SZX9 stereoscopic microscope. All extracted teeth were given the same ID as that of fish samples. A total of 405 teeth of *H. kamiranzovu*, *H. astatodon*, and *H. insidiae* were used for cusp height and width measurements. Among them, only 83 teeth were randomly selected for an outline analysis using the Elliptical Fourier Analysis (EFA): 39 of the bicuspid teeth of *H. kamiranzovu*, 20 bicuspid teeth of *H. astatodon,* and 24 bicuspid teeth of *H. insidiae* (Table 1). The bicuspid teeth extracted from the six individuals of *H. astatodon* were from the IRST group samples. Many of the teeth in these samples were affected by the ethanol in which they were preserved for a long time. We were worried that this few sample size could affect the statistical power of the study. However, it was adequate because the sample size represents a species‐specific morphospace.

The number of teeth examined differed among samples for shape analysis. This depended on the availability of the undamaged teeth on the premaxillary bone of the haplochromine species. These teeth were also used to facilitate the work flow in performing the outline based shape analysis in the SHAPE software. We realized that working with many teeth impeded the SHAPE software to run, and we had to limit the sample size for this analysis which represents a limitation in this study. We ensured that the teeth selected for analysis were distributed across the lake fish sampling locations.

**FIGURE 4:**
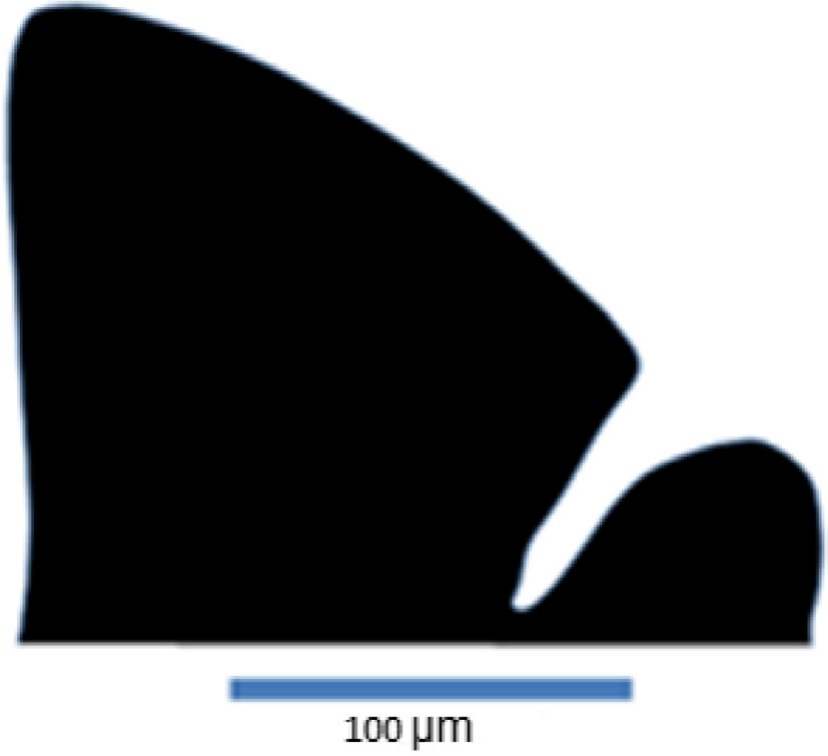
Black and white profile image of the tooth crown of *Haplochromis insidiae* used for automated tracing of the outline and further shape analyses

**FIGURE 5:**
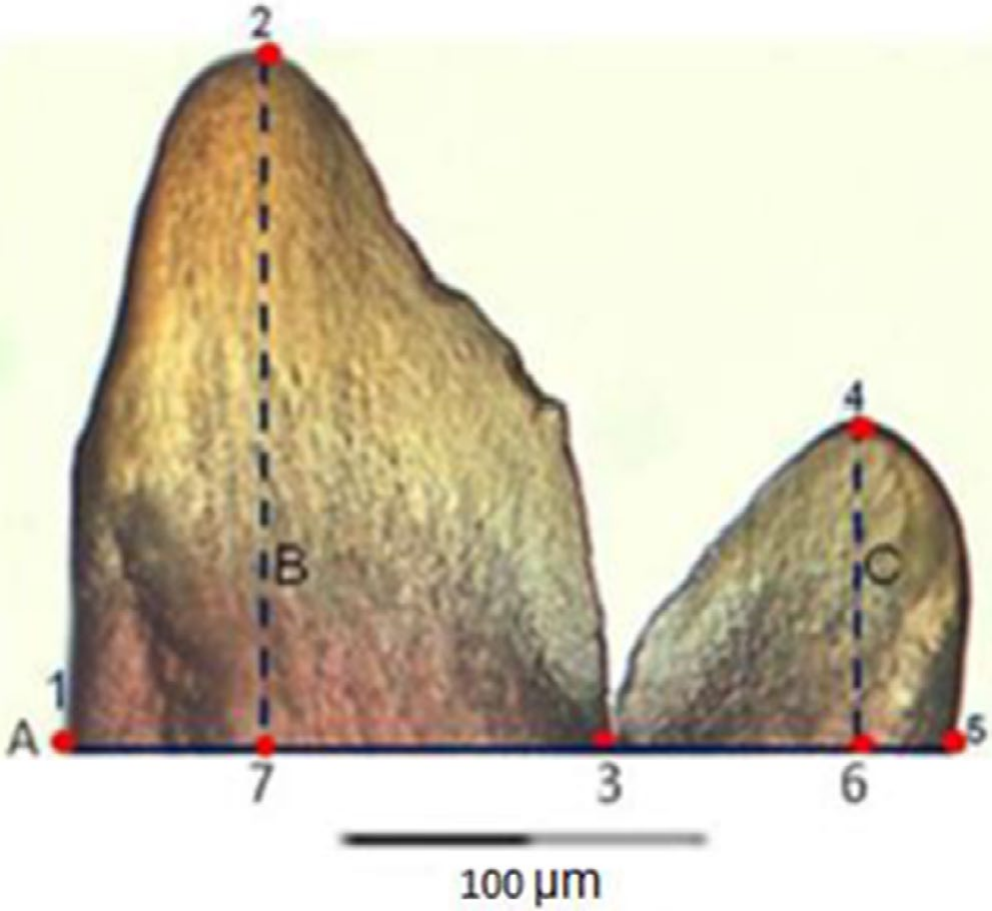
Scheme of the tooth cusps with indication of reference lines and points that were used to calculate height and width of the major and minor cusps (example shows a tooth of *Haplochromis kamiranzovu*). Legend of points: 1 is Posterior edge point of the major cusp in the base line A; 2 & 4 are distal tips of the major cusp and minor cusp, respectively; 3 is indentation point in between the major and minor cusps; 5 is Anterior edge point of the minor cusp in the base A; 6 & 7 are base points of the B and C lines of the major and minor cusps respectively, or is Intersection points of the perpendicular lines B and C with the base line A, used to measure the heights of the major and minor cusps respectively. Legend of reference lines: A—Base line going through points 1 & 3 (representing the maximum width of the major cusp) and going through points 3 &5 (representing the maximum width of the minor cusp). B—Line perpendicular to A and going through the tip of the major cusp (representing the enameloid height of the major cusp); C—line perpendicular to A and going through the tip of the minor cusp (representing the enameloid height of minor cusp)

As teeth were curved along their long axis (Figure 3), the tooth crown was cut at its base on both sides where the enamel–dentine junction (Figure 4) reaches the tooth outline (see points 1, 3, and 5 in Figure 5) under the stereoscopic microscope using a fine surgical blade. This allowed the teeth to be positioned consistently flat on the surface for standardized imaging. However, before the pictures were taken, each tooth crown cusp was immobilized between a glass and cover slip, while submerged in distilled water. The impregnated red color of Alizarin red S solution on these minuscule crown cusps (cleared and stained) facilitated their visibility during manipulation under the microscope. Tooth crown orientation was then standardized by rotating them in such a way that the minor cusp was always positioned at the right side (Figures 4 and 5). Teeth were also positioned so that the cut edge was horizontal, otherwise the software could not continue to proceed. Images were mirrored so that teeth were consistently in the same manner, approximately in the same orientation. Black and white contour outline drawings of the crown cusps were redrawn in Microsoft Power Point (Microsoft) to optimize a correct edge detection. Images in jpeg format were converted into full color bitmap format for uploading in the SHAPE software package for outline analysis (Figure 4). The number of harmonics included in the analysis was 20. This was the maximum number, computed and required to describe the teeth with a relative high degree of precision and closer to the original digitized outline (Crampton, 1995). We did not use the sliding semilandmarks based geometric morphometrics (SSBG) because it can lead to serious statistical and visualization artifacts (Gunz & Mitteroecker, 2013).

### 2.3 Image analysis

Tooth contours were traced using the Chain Coder module of the SHAPE software package ver. 1.3 (Iwata & Ukai, 2002). This involved semi‐automated image processing (converting to black and white images) and contour tracing (describing the outer contour as a chain code). The shape descriptors were obtained after SHAPE models of the tooth specimens were superimposed and scaled to unit size based on the first harmonic (by keeping three of the four descriptors of this harmonic constant). The obtained shape descriptors (elliptic Fourier coefficients—EFCs) were then used for further statistical analyses (Kuhl & Giardina, 1982).

Shape variation across and within species (between sexes, habitats, and regions) was then analyzed by performing a PCA on the variance–covariance matrix of the normalized EFCs. Outlines reflecting the variation explained by the individual PC's were generated using the PrinComp module of the SHAPE package. As the test for normality showed that tooth shape variation was not normally distributed (*p* < .05), the data were subjected to a pair‐wise comparison of a nonparametric MANOVA (npMANOVA) using PAST (Hammer, Harper, & Ryan, 2001) with the Hotelling Bonferroni correction.

To examine species‐specific differences, only bicuspid teeth of the three species were pooled together in both analyses (npMANOVA and PCA (Table 1). However, the shape trait of teeth was consistent in each haplochromine species. This facilitated their identification. For this reason, tooth shape was the only selected trait for their identification with a purely visual tooth inspection using the taxonomic description available. The major assumption in this analysis was that the position of the tooth on the premaxillary bone did not affect the tooth morphology. Using quantitative methods like shape analysis could confirm objectively the species identification. With regard to the size of the crown, we took into account this aspect using traditional measurement of height and width of the major and minor cusps. The variances within and among haplochromine species were separated in statistical analyses (considering males and females individuals within a species and among haplochromine species studied).

Only bicuspid teeth were used to test whether teeth vary between males and females within each species, as indicated in Table 2. The normality test showed that tooth shape variation was not normally distributed among males and females (*p* < .05). Thus, a one‐way npMANOVA on teeth specimens was performed for sex.

**TABLE 2:** Teeth sample size used in pair‐wise comparisons to test for sexual dimorphism in tooth shape

Sex	*Haplochromis kamiranzovu*		*Haplochromis insidiae*		*Haplochromis astatodon*	
	Male	Female	Male	Female	Male	Female
Number of fish	12	7	9	5	3	3
Number of teeth	19	23	17	7	12	8

To check whether tooth shape of the studied species was related to the habitat (as a proxy for ecological niche), we compared teeth from the littoral versus pelagic specimens for *H. kamiranzovu* and *H. insidiae*. *Haplochromis astatodon* was excluded as they were not found in the pelagic zone (Table 3).

**TABLE 3:** Teeth sample size used in pair‐wise comparisons of tooth shape between the fish from the littoral and pelagic zone

Habitat	*Haplochromis kamiranzovu*		*Haplochromis insidiae*	
	Littoral	Pelagic	Littoral	Pelagic
Number of fish	12	4	11	4
Number of teeth	23	6	30	4

Furthermore, for the analysis of geographical variation, teeth of *H. kamiranzovu* and *H. insidiae* were subjected to a south versus north pair‐wise comparison in a one‐way npMANOVA within species across habitats (Table 4). All the above pair‐wise comparisons were subjected to Bonferroni corrections.

**TABLE 4:** Teeth sample size used in pair‐wise comparisons of tooth shape between the fish from the south and north region

Habitat	*Haplochromis kamiranzovu*		*Haplochromis insidiae*	
	South	North	South	North
Number of fish	5	12	9	4
Number of teeth	14	22	19	8

### 2.4 Measurements

Traditional length measurements on teeth crown were used to provide dental metric variations in tooth shape/ tooth size among three studied haplochromine species. The following four measurements were taken on the digital images using ImageJ, ver. 1.44 (Collins, 2007, Figure 5): height of major and minor cusps (measured from point 7 to 2 and from 6 to 4, respectively) and the width of the major and minor cusps (measured from point 1 to 3 and from 3 to 5, respectively). This approach was adopted from premaxillary tooth and crown length and width measurements of cichlids (Casciotta & Arratia, 1993; Dieleman et al., 2015; Wautier et al., 2002).

The height against width measurements on the major cusp of littoral specimens of the three haplochromines species was tested using the nonparametric Kruskal–Wallis rank test to discern differences among the species.

The heights and widths of the major cusp in *H. insidiae* and *H. kamiranzovu* species were tested for normality. They showed that they were not normally distributed (*p* < .05); pair‐wise comparisons between all the groups male versus female, littoral versus pelagic and south versus north of Lake Kivu were then performed for each of the major cusp measurements using a one‐way npMANOVA. The pair‐wise comparisons were also subjected to Bonferroni corrections.

A post hoc test with one‐way ANOVA was done on data from teeth measurements to see which pair‐wise comparisons showed significant differences (Table 5).

**TABLE 5:** Teeth sample size used in pair‐wise comparisons of tooth size between the fish species of female and male, littoral and pelagic, and south and north

Factor	Level	Sample size of teeth	
		*Haplochromis kamiranzovu*	*Haplochromis insidiae*
Sex	Female	124	102
	Male	76	72
Habitat	Littoral	104	87
	Pelagic	96	88
Region of the lake	South	81	123
	North	119	51

## 3 RESULTS

### 3.1 External teeth morphology

**FIGURE 6:**
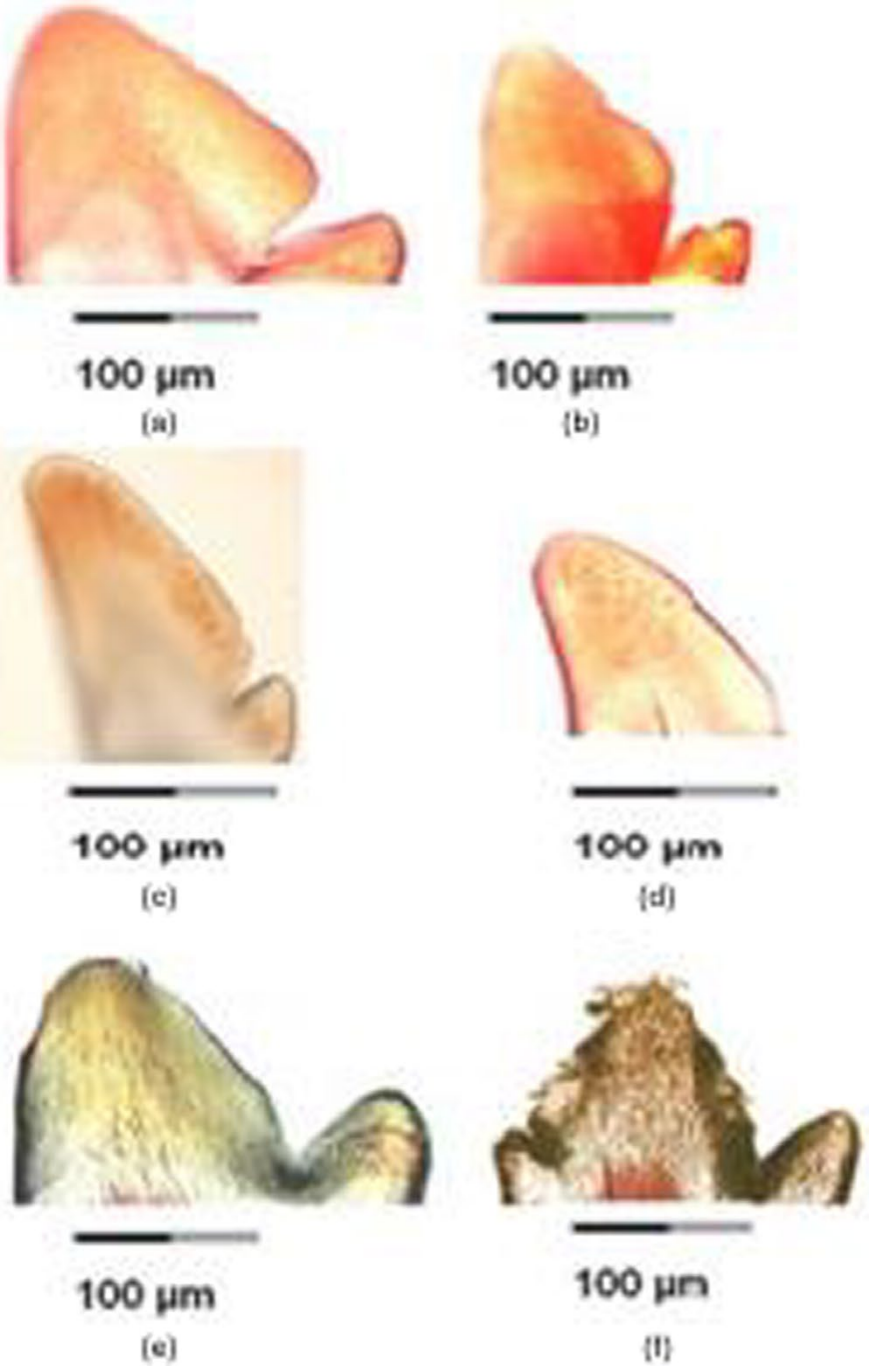
Labial view of the tooth crown in *Haplochromis insidiae* (a, b), *H. astatodon* (c, d) and *H. kamiranzovu* (e, f)

The teeth of *H. insidiae* were all bicuspid (Figure 6a,b). The most distinctive feature was an oblique distal edge (cutting edge formed by the enamel) of the major cusp. In *H. astatodon*, both bicuspid and unicuspid teeth were present (Figure 6c,d). The major cusp of *H. astatodon* was tilted to the left with respect to the crown. Its distal cutting edge was longer than in *H. insidiae* in absolute and relative measurements. In *H. kamiranzovu*, bicuspid and tricuspid teeth were present (Figures 5, 6e,f). In the tricuspid teeth, there was a supplementary minor cusp at the posterior edge of the major cusp (Figure 6f).

### 3.2 Tooth shape in morphospace

**FIGURE 7:**
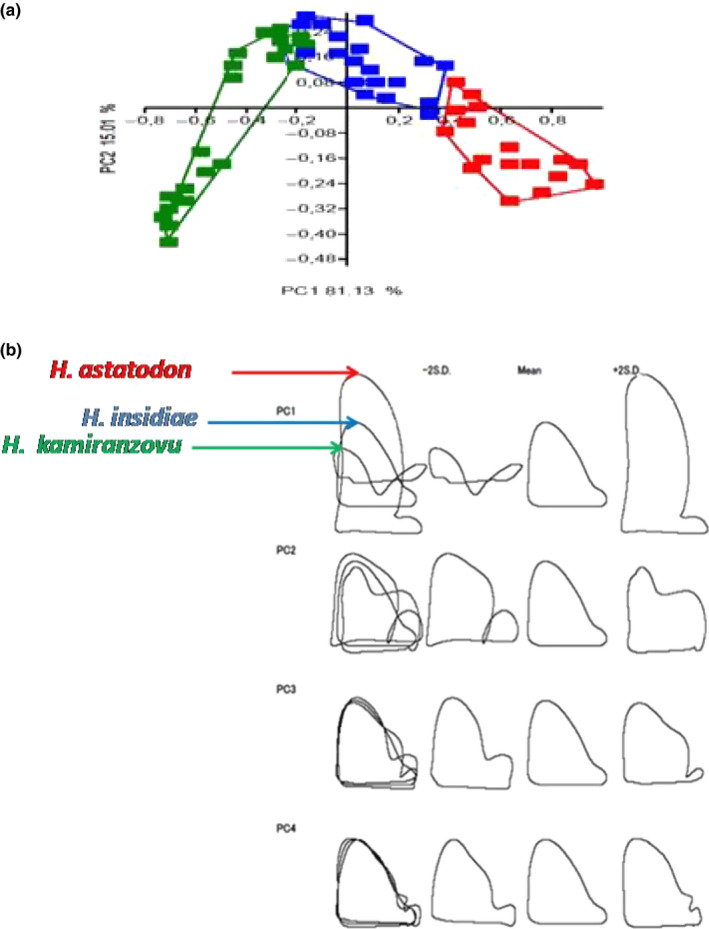
(a) Variations between *Haplochromis astatodon* (red), *H. insidiae* (blue) and *H. kamiranzovu* (green) in major cusp heights and widths, PC 1, as well as in shapes, PC 2 of bicuspid only. (b) Visualisation of the outlines reflecting the mean shape ( centre) and variation (±2× standard deviation) of the only bicuspid data for *H. astatodon*, *H. insidiae,* and *H. kamiranzovu* explained by each of the PC's. The colored arrows represent the superior end of the respective mean major cusp height of each species studied

From principal component analysis, we found significant differences in bicuspid teeth height and width of major cusps, as well as in tooth shape among *H. kamiranzovu*, *H. insidiae,* and *H. astatodon* (*p* < .05, Figure 7a). The results of Wilks' Lambda test and Pillai trace test were 0.06 and 1.36, respectively. The degrees of freedom and *F* values of the above tests were Df1 = 10; Df2 = 146; and the *F* = 44.99 and Df1 = 10; Df2 = 148; and *F* = 32, respectively. This suggests that the majority of the differences between the three species studied is due to differences in height and width of major cusps (Figure 7b and Table 6).

**TABLE 6:** Synopsis of the biometric characters of bicuspid and unicuspid teeth of *Haplochromis astatodon*, *H. insidiae,* and *H. kamiranzovu*

Teeth type of the species	Variable measured	Mean ± *SD*	Range
Bicuspid‐A (*N* = 20)	H	325.84 ± 40.02	254.67–380.03
	W	185.30 ± 16.82	145.33–206.66
	h	37.24 ± 15.91	8.11–65.45
	w	71.42 ± 26.31	21.33–109.46
	H/W	1.75 ± 0.13	1.39–1.96
	h/w	0.52 ± 0.13	0.29–0.89
Unicuspid‐A (*N* = 7)	H	341.82 ± 32.95	293.36–384.08
	W	227.05 ± 23.53	253.33–293.36
	h	‐	‐
	w	‐	‐
	H/W	1.52 ± 0.22	1.28–1.89
	h/w	‐	‐
Bicuspid ‐I (*N* = 174)	H	214.98 ± 43.25	144.02–390.66
	W	173.37 ± 23.12	122.67–253.33
	h	59.94 ± 18.61	22.70–137.39
	w	97.91 ± 14.79	65.34–150.69
	H/W	1.24 ± 0.18	0.84–1.91
	h/w	0.61 ± 0.19	0.20–1.71
Bicuspid ‐K (*N* = 200)	H	199.90 ± 29.35	138.69–292.00
	W	175.63 ± 29.85	122.69–448.00
	h	75.28 ± 19.57	34.89–128
	w	100.02 ± 17.28	53.33–148.02
	H/W	1.14 ± 0.13	0.33–1.50
	h/w	0.75 ± 0.16	0.37–1.29

Note

All the following variables were measured in µm( micro meter). *N* = number of teeth. *H. astatodon* has significantly a longer major cusp height and a longer and larger minor cusp than that of *H. insidiae.* For this also longer than that of *H. kamiranzovu*.

Abbreviations: H, height of the major cusp; h, height of the minor cusp; W, width of the major cusp; w, width of the minor cusp, A: *H. astatatodon*, I: *H. insidiae*, and K: *H. kamiranzovu.*

The SHAPE Software package ver.1.3 performed well using PC2 to visualize tooth shape of the three species. Unfortunately, it failed only to compute the teeth size of *H. kamiranzovu* in PC1 and displayed a very weird size (not a shape) of the species. This might be due to too much intraspecific variability observed in *H. kamiranzovu* teeth.

**FIGURE 8:**
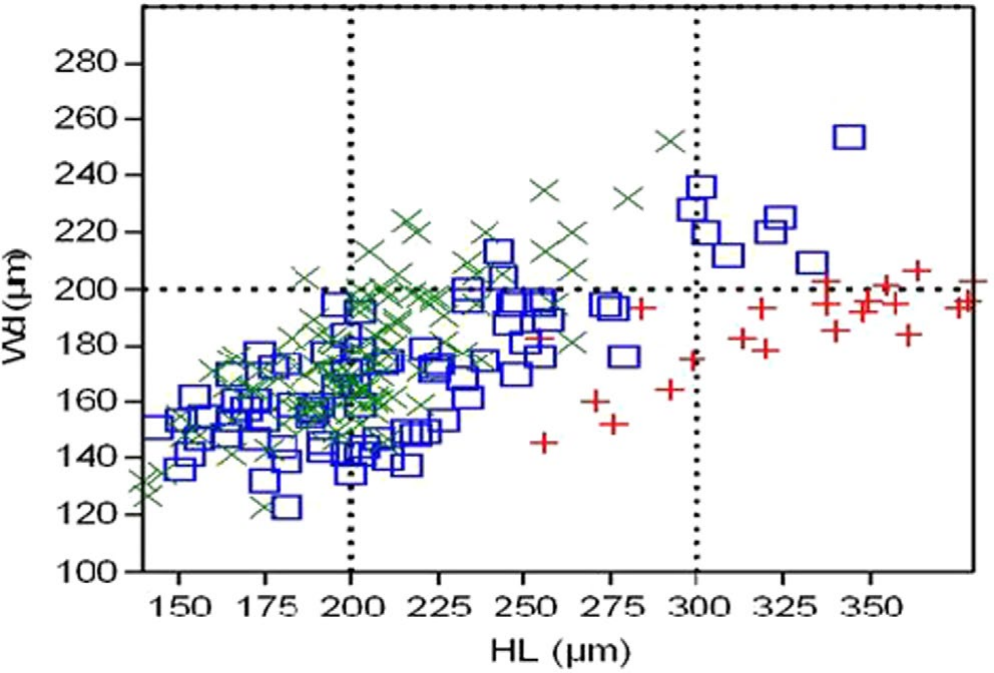
Plot of height against width of the oral teeth per species of *Haplochromis kamiranzovu* (green cross, 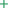 *n* = 104), *H. insidiae* (blue square, 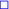 *n* = 87), and *H. astatodon (red cross,* 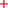 *n* = 22)

The heights and widths of the major and minor cusps of different species are compared in Table 6. The three studied haplochromines species are classified differently according to their teeth shape as described above. Based on PCA results and the above results, the following diagnostic features are suggested for tooth shape differentiation of the three species: the height of the major cusp is larger in *H. astatodon* than that of *H. insidiae,* and they are larger than that of *H. kamiranzovu*. The distal enamel cutting edge of the major cusp of *H. astatodon* is longer than the one of *H. insidiae*. In the latter species, it is shorter than the one of *H. kamiranzovu*. The widths of *H. astatodon* and that of *H. insidiae* are higher than that of *H. kamiranzovu*. The height of the minor cusp in *H. kamiranzovu* is larger than that of *H. insidiae*. In the latter species, it is larger than that of *H. astatodon*. Plotting the major cusp height against the cusp width highlighted the relationship between teeth length differences and haplochromine size as species‐specific (Figure 8).

### 3.3 Sexual shape linked dentition

We uncovered significant sexual differences in tooth shape in *H. insidiae* only (*p* < .05), as well as in height and width of major cusps. The males had longer and wider cusps than females (Table 7).

**TABLE 7:** Size sexual dimorphism in *Haplochromis insidiae* littoral versus pelagic, south versus north measurements of the major and minor cusp of the height and width as well as their ratios

Teeth species for OTU's	Variables measured	Mean ± *SD*	Range
INLF	H	188.98 ± 32.00	164.00–254.68
	W	154.56 ± 11.43	138.72–176.00
	h	69.14 ± 18.11	54.66–113.34
	w	99.18 ± 18.75	72.04–136.05
	H/W	1.21 ± 0.14	1.18–1.46
	h/w	0.70 ± 0.14	0.75–0.94
INLM	H	251.03 ± 4.24	246.67–254.68
	W	195.34 ± 0.76	194.67–196.00
	h	56.41 ± 3.78	50.82–58.80
	w	93.67 ± 3.50	90.66–98.66
	H/W	1.28 ± 5.54	1.26–1.29
	h/w	0.60 ± 1.07	0.56–0.59
INPF	H	194.92 ± 23.51	161.33–254.68
	W	170.64 ± 17.02	142.66–201.35
	h	63.75 ± 20.01	22.70–108.00
	w	96.44 ± 13.97	76.04–121.36
	H/W	1.14 ± 1.38	1.13–1.26
	h/w	0.66 ± 1.43	0.29–0.88
INPM	H	166.94 ± 4.16	162.66–172.04
	W	190.67 ± 2.10	188.00–193.33
	h	62.97 ± 2.20	60.01–65.45
	w	109.63 ± 3.93	105.34–113.34
	H/W	0.87 ± 0.03	0.84–0.91
	h/w	0.57 ± 0.02	0.53–0.60
ISLF	H	198.15 ± 52.93	144.02 390.66
	W	162.27 ± 22.98	122.67 220.00
	h	69.46 ± 16.41	40.08 93.37
	w	98.56 ± 13.49	72–132
	H/W	1.22 ± 2.30	1.71–1.77
	h/w	0.70 ± 1.21	0.55–0.70
ISLM	H	245.10 ± 46.49	172.00–344
	W	182.64 ± 27.99	137.33–253.33
	h	48.45 ± 15.90	29.45–89.33
	w	98.58 ± 17.71	65.34–150.69
	H/W	1.34 ± 1.66	1.25–1.35
	h/w	0.49 ± 0.89	0.45–0.59
ISPF	H	221.25 ± 31.07	156.00–268.00
	W	173.98 ± 23.18	130.67–222.68
	h	59.48 ± 23.90	32.11–137.39
	w	92.68 ± 13.02	69.05–109.40
	H/W	1.27 ± 1.34	1.19–1.20
	h/w	0.64 ± 1.83	0.46–1.25
ISPM	H	223.91 ± 25.91	166.67–273.34
	W	177.74 ± 16.71	152.00–212.10
	h	56.84 ± 8.41	32–76.01
	w	101.36 ± 13.69	66.72–130.67
	H/W	1.26 ± 0.13	1.09–1.48
	h/w	0.56 ± 0.08	0.47–0.74

Note

The males of *H. insidiae* had a larger tooth height and a larger width of the major cusp. Tooth shape of males was also significantly different with more sharp teeth for males than for females. The south of *H. insidiae* had a longer tooth height and a larger width of the major cusp. Their shape was also significantly different with more sharp teeth for south than for north.

Abbreviations: F, female; L, littoral; M, male; N, north; P, pelagic; S, south.

### 3.4 Environmental linked dentition

Our data showed that tooth height and widths of major cusps, as well as tooth shape in *H. insidiae* is also significant between the northern and southern regions of the lake (*p* < .05), (Table 8). The height and width of the major cusp in haplochromine female were consistently higher in the south than in north of the lake, and the same trend was observed in height and width of the minor cusp for haplochromine female being higher in the north than in south. The opposite pattern was found in male of *H. insidiae*, meaning that the height and width of the major and minor cusp were higher in the north than in the south of the lake.

**TABLE 8:** Size sexual dimorphism in *Haplochromis kamiranzovu* littoral versus pelagic, south versus north measurements of the major and minor cusp of the height and width as well as their ratios

Teeth species for OTU's	Variables measured	Mean ± *SD*	Range
KNLF	H	195.19 ± 21.05	141.33–258.69
	W	169.04 ± 20.60	126.66–224.01
	h	83.46 ± 15.82	52–128
	w	107.11 ± 15.24	70.78–148.02
	H/W	1.16 ± 0.12	0.91–1.43
	h/w	0.78 ± 0.13	0.55–1.26
KNLM	H	216.59 ± 35.44	165.35–292.00
	W	189.91 ± 28.54	157.33–252.00
	h	77.73 ± 21.11	46.68–112.03
	w	109.50 ± 16.06	77.37–134.72
	H/W	1.14 ± 0.09	0.94–1.31
	h/w	0.72 ± 0.20	0.43–1.07
KNPF	H	199.17 ± 25.34	150.69–256.00
	W	181.52 ± 51.25	148.00–448.00
	h	82.18 ± 18.94	49.33–114.69
	w	100.93 ± 14.17	74.66–122.67
	H/W	1.13 ± 0.20	0.33–1.39
	h/w	0.82 ± 0.19	0.48–1.29
KNPM	H	197.30 ± 30.21	142.67–264.08
	W	175.70 ± 21.51	130.67–210.93
	h	68.51 ± 24.07	34.89–125.34
	w	91.20 ± 14.96	62.66–122.69
	H/W	1.13 ± 0.16	0.81–1.31
	h/w	0.73 ± 0.18	0.49–1.02
KSLF	H	204.75 ± 36.04	140.00–246.01
	W	179.76 ± 24.26	132.00–220.00
	h	75.36 ± 20.96	46.68–125.34
	w	98.75 ± 17.74	62.66–126.73
	H/W	1.13 ± 0.12	0.93–1.45
	h/w	0.76 ± 0.15	0.55–1.22
KSLM	H	202.41 ± 26.90	153.33–264.00
	W	176.63 ± 23.14	122.69–206.68
	h	69.93 ± 16.89	50.66–112.03
	w	94.62 ± 15.03	70.86–121.34
	H/W	1.15 ± 0.13	1–1.44
	h/w	0.74 ± 0.13	0.51–1.01
KSPF	H	184.36 ± 29.37	138.69–233.36
	W	158.85 ± 17.37	126.69–190.70
	h	63.62 ± 15.38	42.66–97.34
	w	92.42 ± 23.49	53.33–125.36
	H/W	1.15 ± 0.13	0.99–1.50
	h/w	0.70 ± 0.12	0.37–0.87
KSPM	H	194.95 ± 42.96	21.05–292.00
	W	172.10 ± 43.55	17.37–448.00
	h	73.85 ± 22.70	15.38–128
	w	97.32 ± 23.08	14.17–148.02
	H/W	1.11 ± 0.23	0.09–1.50
	h/w	0.74 ± 0.20	0.12–1.29

Note

The males of *H. kamiranzovu* had a larger tooth height and a larger width of the major cusp. Tooth shape of males was also significantly different with more pointy teeth for males than for females. The south of *H. kamiranzovu* had a longer tooth height and a larger width of the major cusp. Their shape was also significantly different with more pointy teeth for south than for north.

Abbreviations: F, female; L, littoral; M, male; N, north; P, pelagic; S, south.

In contrast, no significant differences were found in tooth height and width of major cusps, as well as in tooth shape in *H. insidiae* and *H. kamiranzovu* between the littoral and the pelagic zones of Lake Kivu (*p* > .05, Table 8). Tooth size was significantly different in the littoral versus pelagic populations of *H. insidiae* and *H. kamiranzovu*. Pelagic females of *H. insidiae* had higher height and width of the major cusp with shorter height and width of the minor cusp than the littoral ones. The opposite pattern was found in pelagic males of *H. insidiae.* For *H. kamiranzovu*, most littoral females and males had higher height and width of the major and minor cusp than the pelagic ones (Table 8). An exception was observed in females of *H. kamiranzovu* sampled in the littoral zone of the north.

## 4 DISCUSSION

The purpose of this study was to determine whether there is a diagnostic value of tooth shape in the identification of the Lake Kivu haplochromines. This study used a quantitative analysis on the outlines of teeth. A similar study by Wautier et al., (2002) on fish tooth shape using the same methodology in *Eretmodus cyanostictus* and *E. cf. cyanostictus*, as well as the study by Dieleman et al. (2015) in *Oreochromis hunteri* and *O. korogwe* showed significant differences in tooth shape between species. Referring to the Lake Kivu haplochromines taxonomic study (Snoeks, 1994), it is evident that these species can also be distinguished based on other traits such as morphometric and meristic data. Morphometric and meristic of *H. insidiae* and *H. kamiranzovu* data showed a large overlap. The Elliptic Fourier Analysis (EFA) approach can be used accurately and more objectively to represent gene expression patterns and also assess shape and size variations of gene expression patterns over development of a growing organ (Maeda, Akagi, Onoue, Kono, & Tao, 2019; Martinez‐ Abadias, Mateu, Niksic, Russo, & Sharpe, 2016; Sapala, Runions, & Smith, 2019). In this regard, the use of EFA on tooth shape revealed to be more powerful (Hulme‐Beaman et al., 2019; Van Bocxlaer & Schultheiß, 2010). Thus, tooth shape is a reliable trait to identify haplochromines species in Lake Kivu as suggested by previous observations (Wautier et al., 2002), as it is controlled genetically (Albertson, Streelman, & Kocher, 2003; Kocher, 2004; van Rijssel et al., 2015).

In this study, *H. kamiranzovu* and *H. insidiae* were collected in the same foraging areas of Lake Kivu. Their tooth shape differences are proposed to be a consequence of resource partitioning as suggested by others for cichlid fish (Chinsembu, 2009; Golcher‐Benavides & Wagner, 2019a, 2019b; Ruber et al., 1999). It may be considered as an indicator of different species feeding specialization (Hulsey et al., 2006; Ruber et al., 1999) or a behavior and food types they consume (Kocher, 2004). For example, in Lake Malawi, *Metriacli*ma zebra adults displayed bicuspid dentition that indicates that they are generalist feeders while *Labeotropheus fuelleborni* has tricuspid teeth indicating that they are efficient algal scrapers (Fraser, Bloomquist, & Streelman, 2008). In another study of algae feeding species in Lake Tanganyika, the shape of the jaw teeth differed according to the resources they exploit (Takashi & Koblmüller, 2011). Species of the genus *Petrochromis* were observed to have tricuspid teeth in high density that make a brash‐like structure to comb unicellular algae from filamentous algae on rocks, while species of *Tropheus* were observed to have large bicuspid teeth in the most anterior row that allowed ripping filamentous algae from rocks (Yamaoka, Hori, & Kuratani, 1986). It is commonly known that cichlids tooth shape often resembles their feeding behavior and diet. Thus, tooth shape differences enhance the usefulness of the teeth as a taxonomic tool to distinguish these species (Hulme‐Beaman et al., 2019).

Diagnostic value of tooth shape in the identification of Lake Kivu specimens was also determined by the results of species‐specific differences in tooth shape. These results agree with the findings of Stauffer, Bowers, Kellogg, and McKaye (1997) who reported that tooth shape was one of the morphological characters used to define the genus *Metriaclima* and to discern between the indigenous *Oreochromis hunteri* and the recently introduced *Oreochromis korogwe* in Crater Lake Chala in East Africa (Dieleman et al., 2015). In *H. kamiranzovu*, two bicuspids teeth groups may suggest an existence of two morphotypes within *H. kamiranzovu*. Our results indicated an interspecific tooth shape variation in the three studied Lake Kivu haplochromines as earlier studies showed that tooth shape can vary among species and characterized diverging teeth morphs (Tichy & Seegers, 1999). This result suggests that tooth shape as well as other traits analyzed by Elliptic Fourier is a good predictor to discern species differences at interspecific level (Alvarez & Pérez, 2016; Chuang & Bonhomme, 2019; Oliveira, Panthee, & Silva, 2010).

The PCA reconstructed outlines from the Elliptic Fourier Descriptors (EFDs) showed that the height of the major cusp was the key component in distinguishing the three studied species. As reported by Carlo, Barbeitos, and Lasker (2011), it may be possible to develop diagnostic feature based on the reconstructed images from the EFDs. It is evident that the use of Fourier coefficients yielded a number of characters that could never have been achieved by a purely qualitative description of form as observed in previous study (Carlo et al., 2011; Kenyhercz, Klales, & Kenyhercz, 2013). Tooth shape showed that bicuspid teeth almost differentiated the three species. This result also confirms that the tooth shape is species‐specific trait (Clauss, 2019; Mclean, Helgen, Goodwin, & Cook, 2018; Talebi, Sheidai, & Ariyanejad, 2019; Wautier et al., 2002).

Regarding sexual differences, several studies have been addressed in vertebrates focusing only on tooth size difference (Blanckenhorn, 2005; Garcia & Zuanon, 2019). Few authors have studied sexual differences in tooth shape in general, particularly in cichlids. Sexual difference in tooth shape at intraspecific level existed only in *H. insidiae* among the three studied species. A similar sexual difference in outer tooth shape has been reported for *H. flaviijosephi* (a species from the Jordan Lake), in which adult males had conical and females had bicuspid teeth (Spataru & Gophen, 1985). Similar differences were observed in *H. megalops* in Lake Mwanza Gulf (Witte & Witte‐Maas, 1987). In Lake Kivu, sexual dimorphism in tooth shape was reported previously only in *H. gracilior* (Boulenger 1914) and in *H. graueri* Boulenger (1914), which were among twelve of the fifteen described haplochromines by Snoeks (1994). For both *H. gracilior* and *H. graueri*, it was observed that the occurrence of unicuspid teeth was more abundant in males than in females (Snoeks, 1994). The same author did not observe any sexual dimorphism related to the tooth shape in *H. kamiranzovu* and *H. astatodon* (Snoeks, 1994), and this agrees with our results. No information regarding tooth shape dimorphism in *H. insidiae* was provided by the same author. However, Snoeks (1994) reported sexual dimorphism related to tooth number in the three Lake Kivu haplochromines. The meristic data showing sexual differences in tooth number in fifteen Lake Kivu haplochromines were reported in the published work on taxonomy of Lake Kivu cichlids (Snoeks, 1994).

Tooth shape in *H. insidiae* was associated with geographic differences and not habitat differences. The results indicate that tooth shape variation of *H. insidiae* is influenced by geographic distance suggesting that gene flow may be limited from south to north of the lake or vice versa. Similar results in an Arctic fox, *Vulpes (Alopex) lagopus* showed that tooth shape was significantly correlated with geographic distance (Daitch & Guralnick, 2007). Tooth shape similarity of *H. kamiranzovu* and *H. insidiae* in pelagic zone suggested that food items consumed by these haplochromines species were the same. Similar diet induces similar tooth shape, and this has been reported in other vertebrates (Christensen, 2014). Tooth shape differences observed in *H. insidiae* from littoral south versus littoral north might be linked to its gender differences between male and female individuals. It is known that during spawning, teeth of males may become larger than the ones of females as tools used in territoriality to protect the nests (Auld, Noakes, & Banks, 2019; Johnson et al., 2006). Differences of dental morphology in cichlids have usually been correlated with dietary and behavioral differences (Poll, 1952; Yamaoka et al., 1986) but not with geographic distance. For example, teeth of *Eretmodus* are spatula‐shaped with a slender neck region, those of *Spathodus* are cylindrical with a flattened and truncated crown, and those of *Tanganicodus* are slender and pointed (Ruber et al., 1999). They range from widely spaced and sharply pointed unicuspids in zooplanktivorous and insectivorous species to closely packed tricuspids in algal scrapers. Their tooth shape differences with their associated mtDNA lineages were sampled and linked in particular habitats of Lake Tanganyika. In case of *H. insidiae* in Lake Kivu, tooth shape may be adapted to the regional differences of the Lake Kivu (south vs. north) as food for fish proved to be different between southern and northern parts of the Lake Kivu in terms of food availability of diatoms and cyanobacteria (Isumbisho et al., 2006; Sarmento, Isumbisho, & Descy, 2006). Apparently, one might think that population of *H. insidiae* in the south were isolated by physical barriers to gene flow. The only physical barrier existing in Lake Kivu could be the deeply cut vegetative bays found along the shore line from south to the north. Further research will enable an understanding of the genetic control of tooth shape differences between southern and northern populations of *H. insidiae*. While tooth shape in *H. kamiranzovu* was neither linked to habitat nor geographic differences, it suggests that selective pressure on its tooth shape might be minimal when diet was not significantly different (Daitch, 2008). Tooth shape variation was also observed at interspecific level. This suggests that variation in size and shape of teeth among these haplochromine species may be due to their differences in foraging strategies. This finding is similar to that of Kocher (2004) who observed that tooth morphology was highly correlated with the feeding ecology of cichlid fishes.

Size of the major cusp in the three species was observed to be species‐specific. These results suggest that cusp morphology as well as cusp number, topology, and orientation are species‐specific traits as was previously noticed in different vertebrates (Matalova, Tucker, & Sharpe, 2004). Size of the major cusp in the three studied species was found to be dimorphic. Male individual fish were found to have larger major cusp than female fish. These results are similar to the ones found within each of the breeding habitats of the Eretmodini tribe where male individuals had longer teeth than females (Wautier et al., 2002). Species‐specific size of the major cusp of the three species and their ratios showed taxonomic significance of morphological difference that supports the idea of Wood (1981). He observed that without any prior assumptions about taxonomic groups, the analysis of first cusp mandibular molars measurements, has demonstrated that the major axis of variation separates the pooled sample into morphological subgroups.

Crown height and width differences observed between littoral versus pelagic as well as southern versus northern populations of Lake Kivu support the idea that tooth size trait is an adaptive response to environmental changes or gradients (Albertson et al., 2003; Therry et al., 2019; van Rijssel et al., 2015). Environmental gradients from north to south in the lake probably involve the presence of methane gas and the current extraction of the gas in the northern basin (Boehrer, Tümpling, Mugisha, Rogemont, & Umutoni, 2019) while in south, there is no methane extraction activities.

The observed tooth size changes suggest the existence of phenotypic difference between littoral versus pelagic, and southern versus northern populations as food texture in those habitats might be different that may contribute to the differences in crown cusp size. For instance, *Astatoreochromis alluaudi* that feed on soft and hard foods showed plasticity in phenotype of the teeth of the pharyngeal bone (Huysseune, 1995). The height of the major cusp in the both species *H. kamiranzovu* and *H. insidiae*, in the both regions (south and north) was found to be higher in littoral than in pelagic zone. Similar results were observed in *Haplochromis argens* sp. caught in sand, mud, and rocky habitats of the Lake Victoria, Uganda. The outer‐row teeth were found to be relatively long and slender in those habitats (De Zeeuw et al., 2013).

These shape and size teeth phenotypes suggest species differences in foraging behavior based on available food resources in the lake habitats (Ahirwal, Abidi, Kumar, Singh, & Bavithra, 2019; Golcher‐Benavides & Wagner, 2019a, 2019b). The results of this study suggest resource partitioning between the studied haplochromine species as was observed in other cichlids (Ahirwal et al., 2019). The species demarcation was noticed at the longest teeth. This result is similar to findings of Dieleman et al., (2015) who demonstrated that *Oreochromis* subspecies differences were most pronounced in the longest teeth.

The patterns identified in this study give insight into evolutionary pathway of these haplochromines species and suggest that they may reflect a genetic signal (Torres et al., 2013; Wolpoff, 1971). The EFA results showed that the three species can be distinguished quantitatively based on tooth shape. All taxonomic features mentioned above should be considered to differentiate them. Further revision on Lake Kivu haplochromines taxonomy could consider the tooth shape analysis using the EFA approach to distinguish species.

The current study is the first to address the question of tooth shape analysis using EFA on the three Lake Kivu haplochromines species. The crown size analysis revealed phenotypic variation of the studied haplochromines. Many questions remain to be answered, particularly sexual dimorphism related to each habitat. Further research should also focus on the genetic basis of the observed patterns of dental size as it relates to sexual dimorphism and should be associated with the habitat.

## CONFLICT OF INTEREST

The authors have no conflict of interest to declare.

## AUTHOR CONTRIBUTION

**Philippe Munyandamutsa:** Conceptualization (equal); Data curation (equal); Formal analysis (equal); Funding acquisition (equal); Investigation (equal); Methodology (equal); Project administration (equal); Resources (equal); Software (equal); Validation (equal); Visualization (equal); Writing‐original draft (lead); Writing‐review & editing (lead). **Wilson Lazaro Jere:** Conceptualization (lead); Data curation (lead); Formal analysis (lead); Funding acquisition (supporting); Investigation (lead); Methodology (lead); Resources (supporting); Software (supporting); Supervision (lead); Validation (lead); Visualization (lead); Writing‐original draft (supporting); Writing‐review & editing (supporting). **Daud Kassam:** Conceptualization (lead); Data curation (lead); Formal analysis (supporting); Funding acquisition (lead); Investigation (lead); Methodology (lead); Resources (lead); Software (lead); Supervision (lead); Validation (lead); Visualization (lead); Writing‐original draft (supporting); Writing‐review & editing (supporting). **Austin Mtethiwa:** Conceptualization (lead); Data curation (lead); Formal analysis (lead); Funding acquisition (lead); Investigation (lead); Methodology (lead); Resources (lead); Software (lead); Supervision (lead); Validation (lead); Visualization (lead); Writing‐original draft (supporting); Writing‐review & editing (supporting). 

## Data Availability

Sampling locations and fish morphological data will be archived in dryad https://doi.org/10.5521/dryad.12311.
